# Mitochondrial Optic Atrophy (OPA) 1 Processing Is Altered in Response to Neonatal Hypoxic-Ischemic Brain Injury

**DOI:** 10.3390/ijms160922509

**Published:** 2015-09-17

**Authors:** Ana A. Baburamani, Chloe Hurling, Helen Stolp, Kristina Sobotka, Pierre Gressens, Henrik Hagberg, Claire Thornton

**Affiliations:** 1 Centre for the Developing Brain, Division of Imaging Sciences and Biomedical Engineering, King’s College London, St. Thomas’ Hospital, SE1 7EH London, UK; E-Mails: ana.baburamani@kcl.ac.uk (A.A.B.); chloe.hurling@kcl.ac.uk (C.H.); helen.stolp@kcl.ac.uk (H.S.); pierre.gressens@inserm.fr (P.G.); henrik.hagberg@kcl.ac.uk (H.H.); 2 Perinatal Center, Institute for Clinical Sciences and Physiology & Neuroscience, Sahlgrenska Academy, University of Gothenburg, 41685 Gothenburg, Sweden; E-Mail: kristina.sobotka@gu.se; 3 Inserm, U 1141, 75019 Paris, France; 4 University Paris Diderot, Sorbonne Paris Cité, UMRS 1141, 75019 Paris, France

**Keywords:** mitochondria, OPA1, Oma1, Yme1L, oxygen-glucose deprivation (OGD), hypoxia-ischaemia, neonatal brain injury

## Abstract

Perturbation of mitochondrial function and subsequent induction of cell death pathways are key hallmarks in neonatal hypoxic-ischemic (HI) injury, both in animal models and in term infants. Mitoprotective therapies therefore offer a new avenue for intervention for the babies who suffer life-long disabilities as a result of birth asphyxia. Here we show that after oxygen-glucose deprivation in primary neurons or in a mouse model of HI, mitochondrial protein homeostasis is altered, manifesting as a change in mitochondrial morphology and functional impairment. Furthermore we find that the mitochondrial fusion and cristae regulatory protein, OPA1, is aberrantly cleaved to shorter forms. OPA1 cleavage is normally regulated by a balanced action of the proteases Yme1L and Oma1. However, in primary neurons or after HI *in vivo*, protein expression of YmelL is also reduced, whereas no change is observed in Oma1 expression. Our data strongly suggest that alterations in mitochondria-shaping proteins are an early event in the pathogenesis of neonatal HI injury.

## 1. Introduction

Moderate to severe hypoxic-ischemic encephalopathy (HIE), caused by a lack of oxygen or blood flow to the brain around the time of birth, affects 1.5 in every 1000 live births in the UK and far more in the developing world [1,2,3]. The consequences for babies and parents affected by HIE are devastating; 15%–20% of infants will die in the postnatal period and a further 25% will develop severe and long-lasting neurological impairments [4].

In infants and in animal models of hypoxic-ischemic injury (HI) there is an initial depletion of ATP, phosphocreatine and glucose within the brain followed by a transient recovery to almost physiological levels [5]. However, a second, rapid energy failure facilitates the majority of cell death [6,7,8]. We and others have shown that HI triggers multiple signaling events such as NMDA/AMPA receptor activation, release of reactive oxygen species and increase in intracellular calcium [9,10,11,12]. In addition, cell death after neonatal brain injury is characterized morphologically by a mixed necrotic–necroptotic–apoptotic phenotype depending on time post injury and brain region [10,13,14]. However, data from our lab and others strongly suggest that the common thread linking these diverse mechanisms is mitochondrial dysfunction [15,16].

It is well established that in animal models of neonatal HI, mitochondrial respiration and calcium homeostasis are impaired [17,18,19]. More recently, it was determined that mitochondrial outer membrane permeabilization (MOMP) mediated mitochondrial dysfunction in rodent neonatal HI models [20]. In response to HI, the Bcl-2 family member Bax is activated and translocates to the mitochondria where it complexes and forms a pore with Bak allowing passage of cytochrome c and apoptosis inducing factor (AIF) into the cytosol. Once released AIF and cytochrome c initiate a cascade resulting in activation of caspases, degradation of DNA and ultimately cell death [21]. As such, genetic and pharmacological inhibition of Bax is protective from HI in immature brain [20,22,23]. In addition to Bax-mediated MOMP, mitochondrial ultrastructure is also altered in response to neonatal HI insult [10] and a wide range of mitochondrial morphologies are observed [13]. We therefore hypothesize that such environmental stress may alter mitochondrial dynamics, particularly in the proteins which regulate fission and fusion. Optic Atrophy 1 (OPA1), a dynamin-related guanosine triphosphatase protein, plays a pivotal role in conducting inner-membrane mitochondrial fusion and therefore regulates both mitochondrial cristae junction formation and fusion of distinct mitochondria [24,25,26]. Here we present data analyzing the effect of *in vitro* oxygen-glucose deprivation (OGD) and *in vivo* HI on the processing of OPA1.

## 2. Results

### 2.1. OGD in C17.2 Cells Alters Mitochondrial Function and Morphology

**Figure 1:**
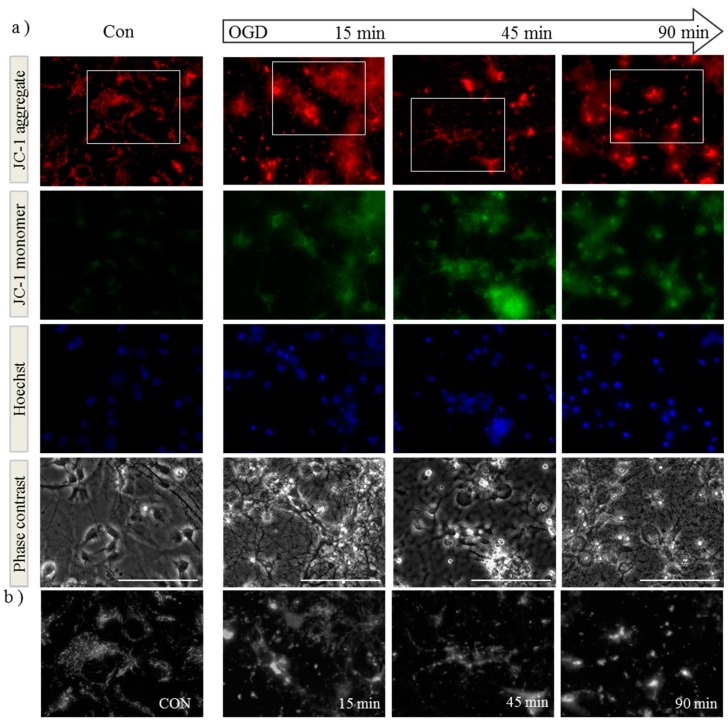

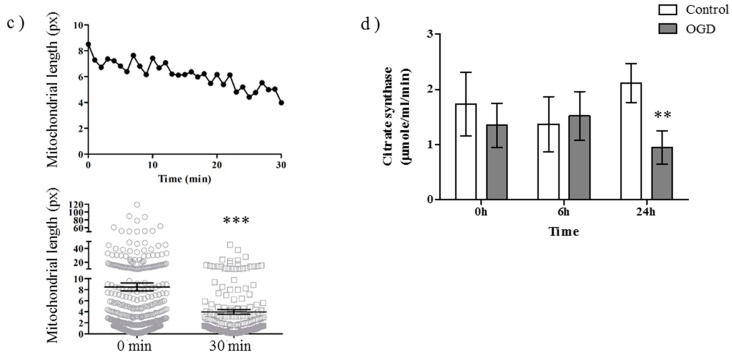
Oxygen-glucose deprivation (OGD) alters mitochondrial membrane potential, morphology and function in primary cortical neurons. (**a**) Primary mouse cortical neurons were loaded with JC-1 dye and Hoechst before exposure to OGD. Cells were imaged live before (Con) and at 15, 45 and 90 min during OGD. Both mitochondrial morphology (as observed in red, top row) and membrane potential (increased green signal, second row) are altered during exposure to OGD. Scale bar represents 100 µm; Figures are representative of three individual experiments: (**b**) Enlargement (3×) of regions defined by white boxes in (**a**). As the experiment progresses, mitochondria morphology appears to alter from tubular structures to round punctate or larger aggregations; (**c**) Primary neurons loaded with JC-1 were imaged every minute during OGD followed by analyses of mitochondrial length. Data shown are an average of 360 mitochondria per time point, and mitochondria from the first and last time points analyzed by student’s *t*-test in the panel below, *** *p* < 0.001; (**d**) Primary neurons were subjected to 90 min of OGD followed by up to 24 h incubation in normal growth medium. Lysates were assayed for citrate synthase activity at time points shown following the insult. Data is shown ± SD, *n* = 4–6 independent litters, determined by two-way ANOVA followed by a Bonferroni *post-hoc* test, ** *p* < 0.01 for interaction and treatment.

OGD is a widely used *in vitro* technique to mimic aspects of cell death observed in *in vivo* HI injury. We performed OGD on mouse primary cortical neurons and examined mitochondrial morphology and membrane potential in live cells throughout the insult using JC-1 dye. Aggregates of JC-1 accumulate in mitochondria in which the membrane potential is maintained, exhibiting red fluorescence, whereas the appearance of diffuse green JC-1 monomers throughout the cell indicates dissipation of membrane potential. Neurons were preloaded with JC-1 before exposure to OGD. Mitochondria were clearly visible in the processes of control cells and generally of uniform size (Figure 1a,b, Con). However after 90 min OGD, we observed an increase in green monomeric JC-1 suggesting impaired membrane potential and altered morphology with both mitochondrial aggregates and rounded puncta (Figure 1a,b, OGD). Similar findings were observed in a recent study of rat cortical neurons exposed to OGD [27], where control mitochondria were found to be tubular and OGD-exposed mitochondria rounded or poorly labelled. In order to quantify these changes, we performed time-lapse imaging on isolated neurons and calculated the changes in mitochondrial length over time. We found a significant decrease in the average mitochondrial length after 30 min of OGD (Figure 1c). After 90 min OGD we returned the cultures to growth medium and analyzed them at subsequent time points for the effect of the insult on mitochondrial health. We found that 24 h post insult, citrate synthase activity was significantly reduced indicating impaired TCA cycle function (Figure 1d). This suggests that neurons which survive the initial insult may subsequently exhibit impaired mitochondrial function.

### 2.2. OPA1 Processing Is Altered after OGD

**Figure 2:**
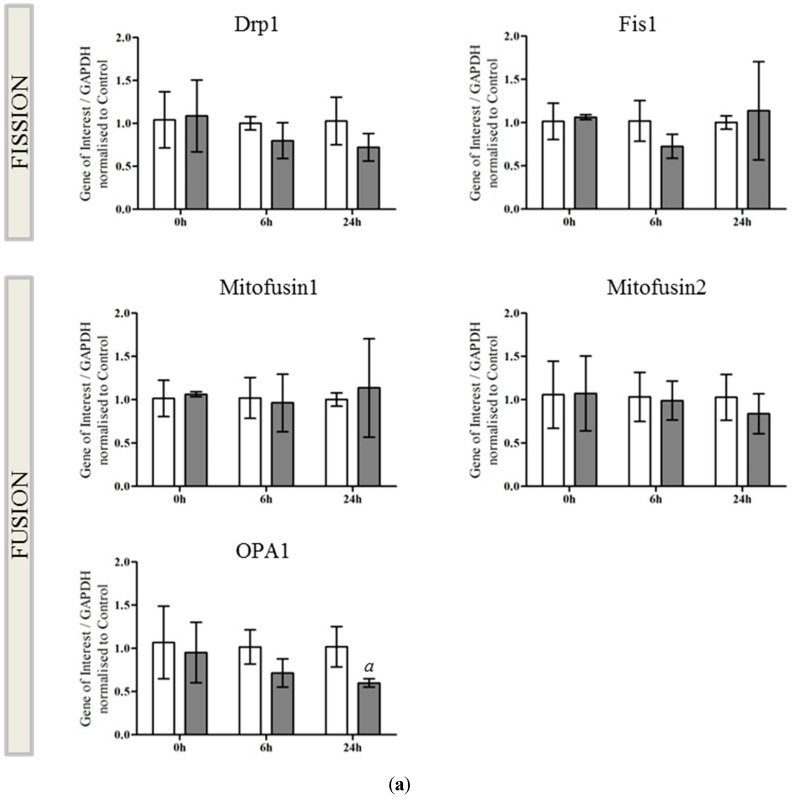

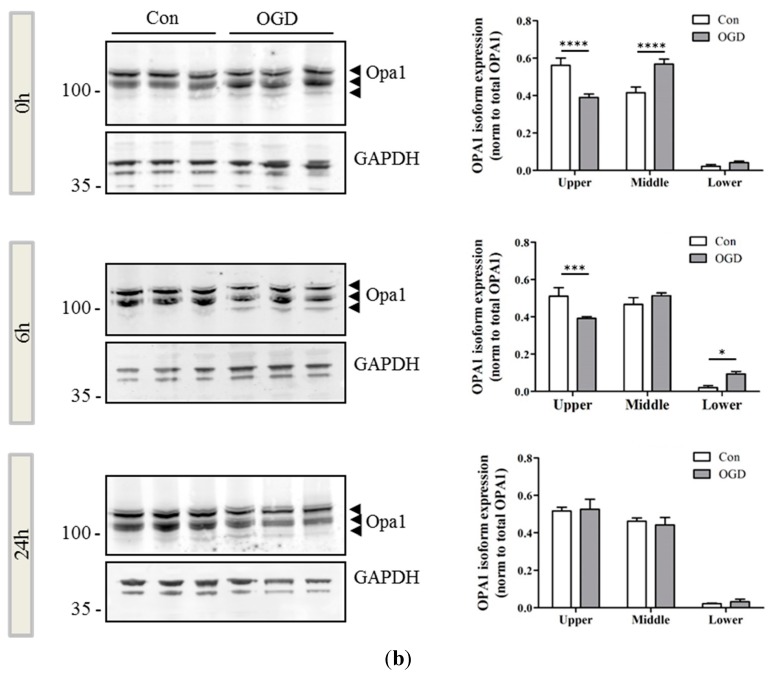
OPA1 processing is altered after OGD (**a**) mRNA generated from control (white bars) and OGD-treated (grey bars) primary neurons was analyzed by qRT-PCR for changes in expression of fission (*Drp1*, *Fis1*) and fusion genes (*Mitofusin 1* and *2*, *OPA1*). There was a small but significant decrease in OPA1 expression in response to OGD exposure (*a* = 0.0296). Mean data shown ± SD (*n* = 4–5 independent litters), significance determined by two way ANOVA; (**b**) Protein lysates were generated from primary neurons either immediately after 90 min OGD (0 h) or following 6 or 24 h recovery. Proteins were resolved by SDS-PAGE and OPA1 analyzed by western blot. Equal protein loading was determined by GAPDH expression. Size distribution of the OPA1 immunoreactivity is expressed as a proportion of total OPA1 (arrowheads). There was a significant increase in the expression of the OPA1 lower band at 0 and 6 h, significance determined by two-way ANOVA for treatment and band. If there was a significant interaction, a Bonferroni *post-hoc* was performed. Data are mean ± SD (*n* = 3 independent litters), * *p* < 0.05, *** *p* < 0.001, **** *p* < 0.0001.

As there was a distinct alteration in mitochondrial morphology in response to OGD, we examined the expression of key genes involved in mitochondrial fission and fusion. Primary neurons were either untreated or exposed to OGD and RNA extracted at 0, 6 and 24 h post insult. Expression of fission genes (*Drp-1*, *Fis-1*) and fusion genes (*Mitofusin 1*, *Mitofusin 2* and *OPA1*) after OGD were compared with expression in control untreated neurons. We found that there was a small but significant decrease in the expression of *OPA1* mRNA comparing treatment groups (Figure 2a, *a* = 0.0296, two-way ANOVA for treatment). To further analyze changes in OGD-mediated OPA1 expression, we generated whole cell lysates from control and OGD-treated neurons and determined OPA1 protein expression by western blot at 0, 6 and 24 h post insult. There was a small decrease in the expression of OPA1 apparent at the 6 h timepoint (Figure 2b). Interestingly, OGD appeared to induce the generation of a smaller band and alter the distribution of remaining OPA1 immunoreactivity. There was a proportional shift towards expression of smaller OPA1 moieties most pronounced at 6 h after OGD, compared with control OPA1 expression. (Figure 2b, arrowheads).

### 2.3. OGD Reduces Yme1L Protein Expression in Primary Neurons

**Figure 3:**
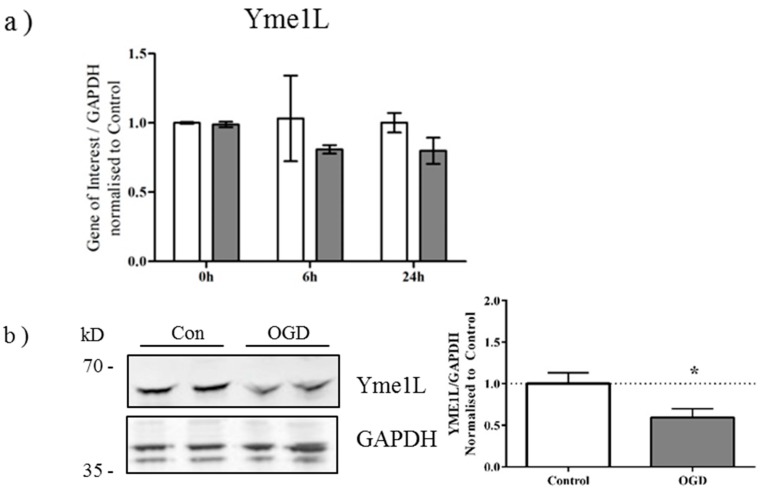

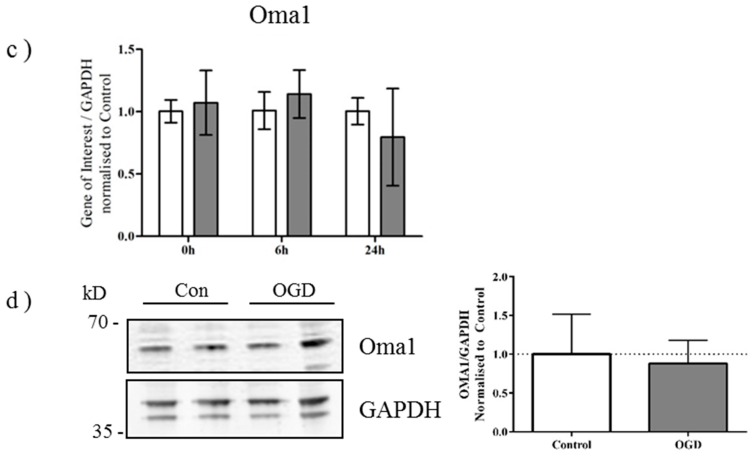
Yme1L protein expression is reduced after OGD (**a**) mRNA generated from control and OGD-treated primary neurons was analyzed by qRT-PCR for changes in expression of Yme1L. Data shown ± SD, *n* = 3–4 independent litters; (**b**) Protein lysates were generated from primary neurons immediately following OGD and analyzed by western blot for expression of Yme1L. Equal protein loading was determined by GAPDH expression which was used for the quantification (right hand panel). Figure is representative of three individual litters, * *p* < 0.05 determined by students *t*-test; (**c**) Oma1 gene expression or (**d**) Oma1 protein expression was determined as above with GAPDH for equal loading. Data was analyzed as above. Figure is representative of three individual litters.

Alternative splicing of OPA1 generates eight isoforms which depending on variant, will contain S1 and S2 cleavage sites, or an S1 site alone [28]. Previous studies have demonstrated that cleavage at S2 by the intermembrane space AAA-protease Yme1L produces a balance of long and short OPA1 products, optimal for OPA1 function [24,29]. When mitochondrial membrane potential is lost, Oma1, a zinc-metalloprotease which resides on the inner membrane, cleaves OPA1 at the S1 site [30,31]. We therefore examined the expression of Yme1L and Oma1 in primary neurons exposed to OGD. Although gene expression of *Yme1L* was not altered significantly in response to OGD (Yme1L *p* = 0.0506; Figure 3a), there was a discernible decrease in Yme1L protein expression at the end of OGD (Figure 3b). Conversely, no changes were apparent for either the gene (Figure 3c) or protein expression (Figure 3d) of Oma1.

### 2.4. Alterations in OPA1 Processing Are Apparent in Vivo after HI

**Figure 4:**
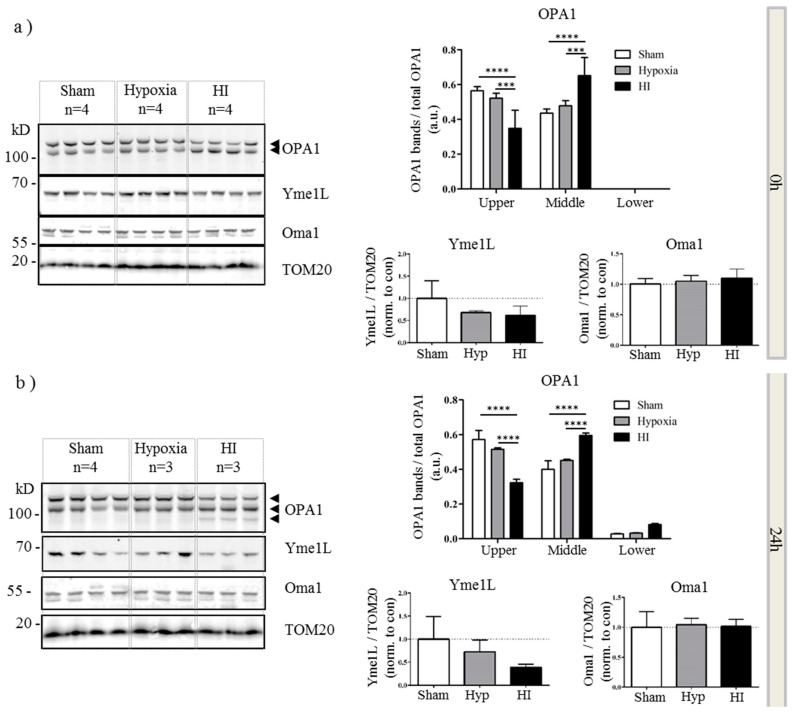

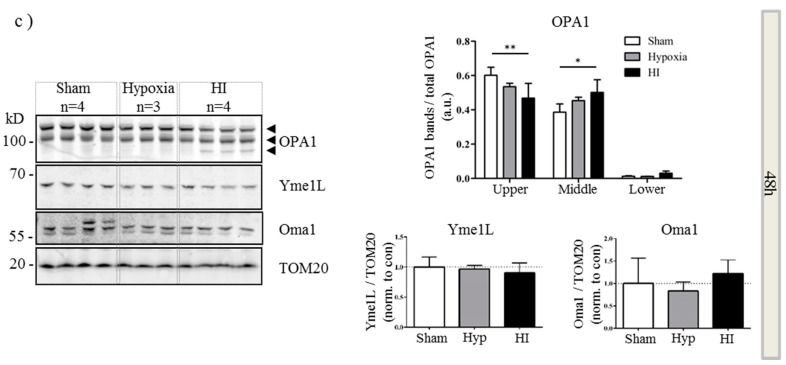
Aberrant processing of OPA1 *in vivo.* Mitochondrial fractions were generated from hypoxic-ischemic (HI), hypoxia alone or sham control mice. Proteins were resolved by SDS-PAGE and analyzed by western blot for OPA1, Yme1L and Oma1 at 0 (**a**), 24 (**b**) and 48 h (**c**) following HI. Tom20 was used as a loading control for mitochondria (bottom panel). Total OPA1 expression was determined by densitometry and OPA1 isoforms or cleavage products expressed as a proportion of that total. Arrowheads indicate alternate forms of OPA1. Mean data shown ± SD. OPA1 significance was determined by two-way ANOVA for band size and treatment followed by a Bonferroni *post-hoc* test * *p* < 0.05, ** *p* < 0.01, *** *p* < 0.001, **** *p* < 0.0001. YME1L and Oma1 data were analyzed with a one-way ANOVA.

Finally we determined if these effects occurred *in vivo* in an animal model of term HI. We used the well-characterized Vannucci HI model in mouse P9 pups, which recapitulates aspects of delayed cell death in human perinatal HI [32,33]. Following unilateral carotid artery ligation, pups are exposed to hypoxia for 75 min before returning to normoxia. This allows both hypoxic (contralateral hemisphere) and hypoxic-ischemic (ipsilateral hemisphere) brain tissue to be sampled from the same animal. Brain tissue was harvested at 0, 24 and 48 h post injury, mitochondrial fractions isolated and OPA1, Yme1L and Oma1 analyzed by western blot (Figure 4a). We found that the bias towards cleaved OPA1 was clearly visible in the hypoxic-ischemic samples from the earliest time point, with a significant decrease of upper band intensity correlating with a significant increase in middle band intensity (Figure 4a, 0 h). Furthermore an additional band of a lower molecular weight was clearly visible in the 24 (Figure 4b) and 48 h (Figure 4c) HI samples. We quantified the upper, middle and lower molecular weight OPA1 bands as a proportion of total OPA1 and observed a significant decrease in the upper band after HI but not hypoxia alone, which was accompanied by a significant increase in the expression of the lower form (Figure 4b). In addition to changes in OPA1, there was a distinct trend towards a decrease in Yme1L expression at 0 (Figure 4a) and 24 h (Figure 4b) which appeared to be resolved by 48 h. Throughout the time course of the experiment, Oma1 expression did not appear to vary (Figure 4a–c). In summary, our data suggest that OGD *in vitro* and HI injury *in vivo* result in cleavage of OPA1 to lower molecular weight forms. This observation correlates with an OGD- or HI-mediated decrease in Yme1L expression.

## 3. Discussion

Dysregulation of energy metabolism is a common feature in a number of human diseases including diabetes, cardiovascular and neurodegenerative disorders [34,35,36]. It is therefore unsurprising the mitochondria are center stage in the development of neonatal brain injury, due to additional high energy demands of the immature brain as it develops [15], and protecting mitochondrial function represents a valid target for future therapeutic intervention. Here we present data suggesting that the homeostasis of inner mitochondrial membrane proteins is disrupted in response to OGD *in vitro* and HI *in vivo* and that alterations in protein function are due largely to post-translational modification.

Although we only identified small changes in OPA1 gene expression, our major finding is that OPA1 is rapidly cleaved to shorter forms in response to hypoxia-ischemia. Shortened forms of OPA1 are reported to occur as a result of environmental stress, resulting in fragmented mitochondria [37,38]. Furthermore, actively altering the balance of long (L-OPA1) and short (S-OPA1) forms may favor mitochondrial fission [39], agreeing with the appearance of small punctate mitochondria apparent during the OGD insult in primary neurons (Figure 1).

OPA1 cleavage occurs due to the actions of the ATP-dependent protease Yme1L and the ATP-independent protease Oma1 [30,31]. Yme1L cleaves OPA1 at the S2 site and subsequent products remain fusion-competent [24,29]. However, loss of mitochondrial membrane potential results in cleavage at OPA1 S1 site by Oma1 and generation of fusion-incompetent mitochondria [31]. Our results (Figure 1) and those of others [27,40] suggest that exposure of primary neurons to OGD induces a decrease in mitochondrial membrane potential coupled with changes in mitochondrial morphology (Figure 1, Con *vs*. OGD).

Concomitantly, post insult, we observed a decrease in Yme1L protein expression *in vitro* (Figure 3) and *in vivo* (Figure 4). It is well established that in animal models of HI and *in vitro*, the insult induces a rapid depletion of cellular ATP [8,33,41]. Yme1L activity is ATP-dependent and more recently, its expression was found to be reduced following oxidative stress [42]. These authors also identified Oma1 as an ATP-independent protease which regulated Yme1L expression; resistance to Oma1-mediated Yme1L degradation was conferred by ATP binding. Both our *in vitro* and our *in vivo* results are in line with these findings suggesting that loss of Yme1L may sensitize cells to oxidative stress through dysfunctional mitochondrial dynamics [43]. However, further work is required to determine whether disrupting the balance of Yme1L and Oma1 is critical in neonatal hypoxic-ischemic brain injury.

In the development of neonatal brain injury in response to HI insult, we and others have documented the induction of apoptosis resulting in cytochrome c release from permeabilized mitochondria [13,44,45,46]. However, the majority of cytochrome c is held within the cristae, relying on cristae reconfiguration to allow it access to move into the intermembrane space once apoptosis is induced. In addition to its role in fusion, OPA1 controls the integrity of the cristae [25] and inhibition of OPA1 leads to cristae disorganization [26]. Our data suggest the possibility that OPA1 processing may be an early but key step in the propagation of neuronal cell death induced by neonatal hypoxic-ischemic injury. Interestingly, during revision of our manuscript, Sanderson and colleagues identified release of cytochrome c and appearance of degraded OPA1 in the cytosol in response to OGD/reperfusion in primary neurons [47] providing further evidence for our hypothesis. Our future studies will therefore center on the roles of Yme1L and Oma1 in the regulation of OPA1 in order to highlight whether prevention of such cleavage is neuroprotective in neonatal hypoxic-ischemic injury.

## 4. Experimental Section

### 4.1. Research Ethics Statement

All animal use was in accordance with local rules (King’s College London, Animal Welfare and Ethical Review Board, London, UK) and with the regulations and guidance issued under the Animals (Scientific Procedures) Act (1986) covered by Home Office personal and project licenses.

### 4.2. Primary Cortical Neuron Preparation

C57/Bl6 pregnant mice (Charles Rivers, Margate, UK) at embryonic day 13.5–15.5 were killed by schedule 1 methods. Embryonic cerebral cortices were dissected and tissue from the same litter pooled. Primary cortical neurons were prepared as described previously [48], plated at a density of 2 × 10^6^ cells/6 cm plate and maintained in neurobasal medium (Life Technologies, Paisley, UK) containing B27^®^ (Life Technologies), l-Glutamine (Sigma, Gillingham, UK) and Streptomycin/AmphotericinB (Life Technologies,). Cultures were maintained at 37 °C, 5% CO_2_.

### 4.3. Neonatal Hypoxia-Ischemia

C57/Bl6 mice (Charles River) at postnatal day 9 (P9) were subjected to unilateral hypoxia-ischemia, essentially according to the Rice–Vannucci model that results in a focal ischemic injury allowing for comparison between an HI (ipsilateral) and hypoxia alone (contralateral) hemisphere [32,33]. Briefly, mice were anesthetized with isoflurane (4.5% for induction and 2% for maintenance) in a mixture of nitrous oxide and oxygen (1:1), with the duration of anesthesia being <5 min per pup. The left common carotid artery was isolated and ligated. Pups recovered for 1–2 h in the parent cage. Litters were placed in a chamber with a humidified hypoxic gas mixture (10% oxygen in nitrogen, 36 °C) for 75 min. Sham control mice were not subjected to surgery or hypoxic chamber. After hypoxic exposure, pups were returned to their biological dams until the conclusion of the experiment. Both females and males pups were used and treatment groups contained pups from at least 3 independent litters.

### 4.4. Oxygen-Glucose Deprivation (OGD) of Primary Neurons

Primary neurons were cultured for a minimum of 6 days (DIV6) prior to treatment then loaded with JC-1 (5 µM; Life Technologies) and Hoechst (10 µg/mL; Sigma) for 30 min, 37 °C, 5% CO_2_. Growth medium was replaced with de-gassed, glucose-free, Neurobasal-A medium (Life Technologies) and culture plates mounted on the EVOS/Chamlide microscope (Life Technologies) where they were maintained at 37 °C in a 95% N_2_/5% CO_2_ environment for the duration of the OGD. Following OGD, medium was replaced with standard Neurobasal medium (containing additions described above) and cultures returned to 5% CO_2_ incubation. For control plates, medium was replaced with standard Neurobasal media at the start of the OGD plates and replaced again with media after 90 min. Cells were collected at 0, 6 or 24 h following treatment.

### 4.5. MTT Assay

Primary cortical neurons were assayed for mitochondrial reductase activity using the MTT (3-(4,5-Dimethylthiazol-2-yl-)-2,5-diphenyl-2H-tetrazolium bromide; Sigma) assay as described previously [49].

### 4.6. Citrate Synthase Assay

Primary neurons were lysed in CelLytic MT Cell Lysis Reagent (Sigma), protein concentration determined by BCA assay (Thermo Scientific, Loughborough, UK) and were assayed for enzymatic activity using Citrate Synthase Assay Kit (Sigma) according to manufacturer’s instructions. Eight micrograms of total protein was used per reaction on a 96-well plate and independent samples were measured in duplicate. Absorbance was read at 412 nm on a SPECTROstar Nano plate reader using MARS analysis software (BMG Labtech, Aylesbury, UK). Baseline absorbance was measured every 30 s for 5 min, and following addition of oxaloacetic acid, total activity was measured every 10 min for 60 min.

### 4.7. qRT-PCR

RNA was harvested using the Direct-zol RNA MiniPrep kit (Zymo Research, through Cambridge Biosciences, Cambridge UK) as per manufacturer’s instructions. Total RNA (100 ng) was analysed using Taqman gene expression assays and RNA-to-CT kit (Life Technologies) on a StepOneplus Cycler (Life Technologies). Data were normalized to the expression of GAPDH and to controls for each time point using the 2^−^^ΔΔ*C*t^ method [50]. The following primer pairs were used: *GAPDH* (Mm99999915), *DRP1* (Dmn1; Mm01342903), *Fis1* (Mm00481580), *Mitofusin1* (Mm00612599), *Mitofusin2* (Mm00500120), *OPA1* (Mm01349707), *Oma1* (Mm01260328) and *Yme1L* (Mm00496843) from Life Technologies.

### 4.8. Subcellular Fractionation

For preparation of mitochondrial fractions, mice were sacrificed at 0, 24 and 48 h post HI and brains rapidly dissected into ice-cold subcellular fractionation buffer (250 mM sucrose, 20 mM HEPES pH 7.4, 10 mM KCL, 1.5 mM MgCl_2_, 1 mM EDTA, 1 mM EGTA, 1 mM DTT, 1× protease inhibitor cocktail). Samples were homogenized in a 2 mL dounce homogenizer on ice, passed through a 27 G needle and incubated on ice for 20 min. The suspension was centrifuged (720× *g*, 5 min) to remove the nuclear fraction and any unbroken cells. The resulting supernatant was centrifuged (15,000× *g*, 15 min) to obtain a mitochondrial pellet and a cytosolic supernatant.

### 4.9. Western Blot

For protein analysis primary neurons were lysed in HEPES buffer A (50 mM HEPES (pH 7.5), 50 mM sodium fluoride, 5 mM sodium pyrophosphate, 1 mM EDTA, +1× protease inhibitors (Sigma)) containing 1% (*v*/*v*) Triton X-100 (Sigma). Protein from cell lysates (50 µg) and mitochondrial fractions from brain lysates (30 µg) were resolved on 4%–12% gel (*w*/*v*) NuPAGE BisTris gels in MOPs buffer (ThermoFisher Scientific, Hemel Hempstead, UK) and transferred to polyvinylidene fluoride membrane (PVDF, Millipore, Beeston, UK) in NuPAGE transfer buffer (Life Technologies). Membranes were blocked in 5% skim-milk in Tris-buffered saline with 0.1% Tween 20 (TBS-T) and incubated overnight with the following antibodies: anti-mouse OPA1 (1:1000, Cat# 612606, BD Biosciences, Oxford, UK), anti-rabbit YME1 (1:1000, Cat# 11510-1-AP, ProteinTech, Manchester, UK), anti-rabbit OMA1 (for primary neurons; 1:1000, Cat# NBP1-56970, Novusbio, UK and for mitochondrial fractions from brain lysates; 1:750, Cat# 17116-1-AP, ProteinTech), anti-mouse GAPDH (1:2000, Cat# G8795 Sigma) and for mitochondrial fractions Tom20 (1:750, Cat# sc-11415, Santa Cruz Biotechnology, Wembley, UK). Secondary antibodies: Li-cor IRDye Goat anti-rabbit 800 or Goat anti-mouse 680 secondary antibodies were used at 1:10,000 and incubated for 2 h at room temperature. Membranes were imaged on a Li-Cor Odyssey Infrared Imaging System (Li-Cor Imaging Biotechnology UK Ltd., Cambridge, UK) using the manufacturer’s Image Studio software for band quantification.

### 4.10. Data Analysis

Data are expressed as mean ± SD. All primary neuronal culture experiments were performed on 3–6 independent litters as stated in the figure legends. Mitochondrial length was analyzed using Squassh (segmentation and quantification of subcellular shapes) software, part of the Mosaic toolkit in Image J [51]. All statistical analyses were performed using GraphPad Prism 6 Software (GraphPad Software, San Diego, CA, USA). All data were assessed for normality. Data was assessed either by a Student’s *t*-test or for multiple conditions, with a two-way ANOVA. If significant, a Bonferroni *post-hoc* analysis was conducted. Analyses used are detailed in table and figure captions. * *p* < 0.05, ** *p* < 0.01, *** *p* < 0.001, **** *p* < 0.0001.

## 5. Conclusions

We have determined that in primary neurons, mitochondrial membrane potential, morphology and function are impaired in response to OGD. Furthermore, we present the first data suggesting that in a mouse model of neonatal HI, the expression of mitochondrial shaping proteins, such as OPA1 and Yme1L, are altered; *in vitro* and *in vivo*, OPA1 is cleaved to shorter forms and Yme1L expression is reduced. Further studies are required to determine the molecular pathways regulating these events.
